# The significance of serum HMGB1 level in humans with acute paraquat poisoning

**DOI:** 10.1038/s41598-019-43877-1

**Published:** 2019-05-15

**Authors:** Feng Chen, Zuolong Liu, Wei Li, Dan Li, Bailing Yan

**Affiliations:** 1 Dermatology Department, China-Japan Union Hospital of Jilin University, 126Xiantai Street, Changchun, 130033 Jilin Province People’s Republic of China; 2 Department of Emergency, The First Hospital of Jilin University, 71Xinmin Street, Changchun, 130021 Jilin Province People’s Republic of China; 3 Department of Respiratory Medicine, The First Hospital of Jilin University, 71Xinmin Street, Changchun, 130021 Jilin Province People’s Republic of China

**Keywords:** Biophysical chemistry, Prognostic markers

## Abstract

High-mobility group box 1 (HMGB1) mediates acute lung injury in a mouse model of paraquat poisoning. However, published reports showing a clinically relevant association between HMGB1 and paraquat exposure are lacking. The objective of the present study was to investigate the potential role of serum HMGB1 level as a prognostic marker of mortality in patients with paraquat poisoning in a clinical setting. This retrospective observational cohort study included a convenience sample of 92 patients with acute paraquat poisoning admitted to the emergency room (ER) of The First Hospital of Jilin University between January 2014 and December 2016. Baseline serum HMGB1 levels and other laboratory parameters were measured on admission. Cumulative incidence of mortality during the first 30 days after admission was 50% (n = 46/92). Serum HMGB1 levels were higher in fatalities than survivors (*P* = 0.015), 30-day mortality increased with increasing baseline serum HMGB1 level (*P* < 0.001), and higher serum HMGB1 levels were associated with an increase in 30-day mortality on Kaplan-Meier analysis. Multivariate Cox regression analysis identified baseline serum HMGB1 levels, white blood cell count, and serum lactic acid levels as independent prognostic markers of 30-day mortality. These data suggest that serum HMGB1 levels measured on admission to the ER are an independent predictor of 30-day mortality in patients with acute paraquat poisoning.

## Introduction

Paraquat is an organic compound containing the active ingredient 1,1′-dimethyl-4,4′-bipyridinium dichloride that is widely used as a herbicide. Globally, paraquat is a leading cause of lethal poisoning, particularly in developing countries^1–6^. In fact, oral ingestion of paraquat for deliberate self-harm has become a severe public health problem in several countries^1–4^.

Currently, oxidative stress is implicated as the mechanism underlying the toxic effects produced by paraquat. Biotransformation of paraquat in cells results in the generation of reactive oxygen species (ROS) and apoptosis-related molecules, which cause multiple organ dysfunction in humans and animals, mainly involving the lung, kidney, heart, liver, and central nervous system.

Recent evidence suggests that the molecular mechanisms underlying paraquat toxicity include the release of damage-associated molecular patterns, including high-mobility group box 1 (HMGB1), a chromatin-binding protein that can be secreted by necrotic or inflammatory cells. Previously, we reported that a signaling cascade involving HMGB1, toll-like receptor 4 (TLR4), interleukin 23 (IL23), and IL17A mediated acute lung injury (ALI) in a mouse model of paraquat poisoning^7^. These findings suggest that targeting the HMGB1-TLR4-IL23-IL17A axis represents a potential therapeutic strategy for treating paraquat poisoning. However, published reports showing a clinically relevant association between HMGB1 and paraquat exposure are lacking. Therefore, the objective of the present study was to investigate the potential role of serum HMGB1 level as a prognostic marker of mortality in patients with paraquat poisoning in a clinical setting.

## Methods

### Study subjects

This study was approved by the ethics committee of The First Hospital of Jilin University and was conducted in accordance with the Declaration of Helsinki. Written informed consent was obtained from all patients on admission to the emergency room (ER).

This retrospective observational cohort study included a convenience sample of patients presenting to the ER of The First Hospital of Jilin University between January 2014 and December 2016 with acute paraquat poisoning, defined as an oral intake of 2–200 mL paraquat during the prior 24 hours. Exclusion criteria were: (1) diabetes mellitus, hypertension, renal failure, or malignancy; (2) >24 hours since oral intake of paraquat; or (3) incomplete follow-up data. Hospitalized patients were followed up to death. Discharged patients were followed up to death or for 30 days. Twenty healthy subjects were enrolled to investigate baseline HMGBI levels in a group of healthy controls.

### Sample collection and biochemical analyses

Each patient provided a peripheral venous blood sample (5 mL) during the first 24 hours after admission to the ER. Sera were isolated, frozen, and stored at −80 °C until use. Serum HMGB1 levels were measured using an enzyme-linked immunosorbent assay kit according to the manufacturer’s instructions (Yanhui Biotech, Shanghai, China).

Baseline hematological and laboratory parameters, including white blood cell (WBC), neutrophil, lymphocyte, and platelet counts, hemoglobin, prothrombin time, total bilirubin, albumin, alanine aminotransferase (ALT), aspartate aminotransferase (AST), creatinine, serum amylase, troponin I (cTnI), pH, partial pressure of carbon dioxide (PaCO2), and partial pressure of oxygen (PaO2) were measured using a Hitachi 7600 Clinical Analyzer (Hitachi, Tokyo, Japan), Sysmex CA-7000 System (Sysmex, Kobe, Japan), and Sysmex XE-5000 (Sysmex)^8^, according to standard protocols.

### Statistical analysis

Statistical analyses were conducted using SPSS, v24 (SPSS, Chicago, IL, USA). Values are expressed as mean ± standard error of the mean (SEM) for normally distributed data or median and interquartile ranges for non-normally distributed data. One-way analysis of variance (ANOVA) and the Kruskal–Wallis H test or Mann–Whitney U test were performed to evaluate between-group differences. Chi-squared test was conducted to evaluate between-group differences stratified by gender and in 30-day mortality. A Kaplan-Meier curve was constructed to describe survival. Univariate and multivariate Cox regression analyses were performed to identify predictors of 30-day mortality; results are summarized as hazard ratios with corresponding 95% confidence intervals (CIs). Variables with a *P* value < 0.1 on univariate analysis were included in the multivariate analysis. *P* < 0.05 was considered statistically significant.

## Results

### Study population

This study enrolled 304 patients with an oral intake of 2–200 ml paraquat. A total of 212 patients were excluded. Of these, 21 patients had diabetes mellitus, 29 patients had hypertension. 19 patients had renal failure, 22 patients had malignancy, 104 patients had inadequate follow up data, and >24 hours had passed since the oral intake of paraquat in 17 patients. The remaining 92 patients were included in the final analysis. These patients had a median age of 33.5 years (range: 13–77 years) and 47 were female and 45 were male.

### Baseline demographic and clinical characteristics

Patients were divided into tertiles according to their baseline serum HMGB1 levels: tertile 1, HMG1:0.41–6.67 μg/mL; tertile 2, 7.12–11.36 μg/mL; tertile 3, 11.56–15.34 μg/mL. Baseline demographic and clinical characteristics of included patients stratified by HMGB1 tertile are summarized in Table 1. Baseline WBC and neutrophil counts and serum lactic acid levels significantly increased across tertiles from lowest to highest serum HMGB1 levels, while arterial pH decreased.

**Table 1:** Baseline characteristics of patients stratified by serum HMGB1 tertile (n = 92).

Variable	Tertile 1 (n = 30)	Tertile 2 (n = 30)	Tertile 3 (n = 32)	*P* value
Age	37.5 (27.5–48.0)	30.0 (23.75–39.25)	33.5 (25.0–48.25)	0.162
Gender(male/female), n	11:19	15:15	19:13	0.2
Time from ingestion to ED (hrs.)	6.0 (3.75–11.25)	6.5 (4.0–18.25)	6.0 (4.0–8.75)	0.592
MAP (mmHg)	89.67 (86.25–96.75)	86.67 (82.17–96.17)	90.17 (86.67–100.33)	0.227
Respiratory rate (breaths/min)	20 (16–20)	20 (18–20)	20 (18–23)	0.184
pH	7.41 (7.39–7.45)	7.41 (7.39–7.44)	7.39 (7.32–7.41)	0.006
PaCO_2_ (mmHg)	37.5 (31.0–41.0)	39.5 (34.5–40.3)	36.0 (21.341.0)	0.265
PaO_2_ (mmHg)	88.0 (75.0–101.0)	93.0 (77.3–103.0)	89.0 (71.3–105.3)	0.642
lactic acid (mmol/L)	1.0 (0.7–2.0)	1.35 (0.6–3.4)	4.1 (2.5–11.7)	<0.001
WBC (×10^9^/L)	10.94 (7.5–15.4)	10.6 (8.5–17.4)	17.0 (9.3–22.0)	0.042
Neutrophil (×10^9^/L)	9.2 (5.9–13.9)	9.1 (6.7–15.1)	14.3 (7.5–20.5)	0.034
Lymphocyte (×10^9^/L)	0.9 (0.6–1.8)	1.1 (0.7–1.8)	1.3 (0.8–1.6)	0.947
Hg (g/L)	141.5 (117.8–149.8)	145.0 (129.8–155.3)	152.0 (134.0–158.8)	0.077
Platelet (×109/L)	254.4 ± 87.56	212.2 ± 81.75	227.5 ± 67.64	0.119
BUN	5.2 (3.9–6.6)	4.8 (3.2–6.2)	5.1 (4.1–7.3)	0.411
Cr	62.0 (48.6–117.6)	62.4 (53.8–112.7)	87.3 (59.3–133.5)	0.101
Albumin (g/dL)	41.5 (39.7–46.2)	43.6 (41.0–6.1)	4.4 (40.3–47.6)	0.473
total bilirubin	13.0 (9.5–15.2)	15.6 (13.0–24.6)	17.5 (10.5–23.4)	0.058
ALT (U/L)	24.5 (17.3–34.3)	26.0 (19.7–36.3)	26.2 (20.9–45.0)	0.164
AST (U/L)	19.8 (12.6–27.0)	19.1 (13.0–34.4)	23.0 (19.4–43.2)	0.128
cTnI	0.012 (0.01–0.02)	0.012 (0.01–0.02)	0.012 (0.012–0.028)	0.285
serum amylase	72.0 (52.0–105.8)	71.5 (38.5–100.8)	79.4 (55.5–120.0)	0.651

MAP, mean arterial pressure; PaCO_2_, partial pressure of carbon dioxide; PaO_2_, partial pressure of oxygen; WBC, white blood cell; Hg, hemoglobin; CK, creatine kinase; BUN, blood urea nitrogen; AST, aspartate transaminase; ALT, alanine transaminase.

### Comparison of the serum HMGB1 level between fatalities and survivors

**Figure 1:**
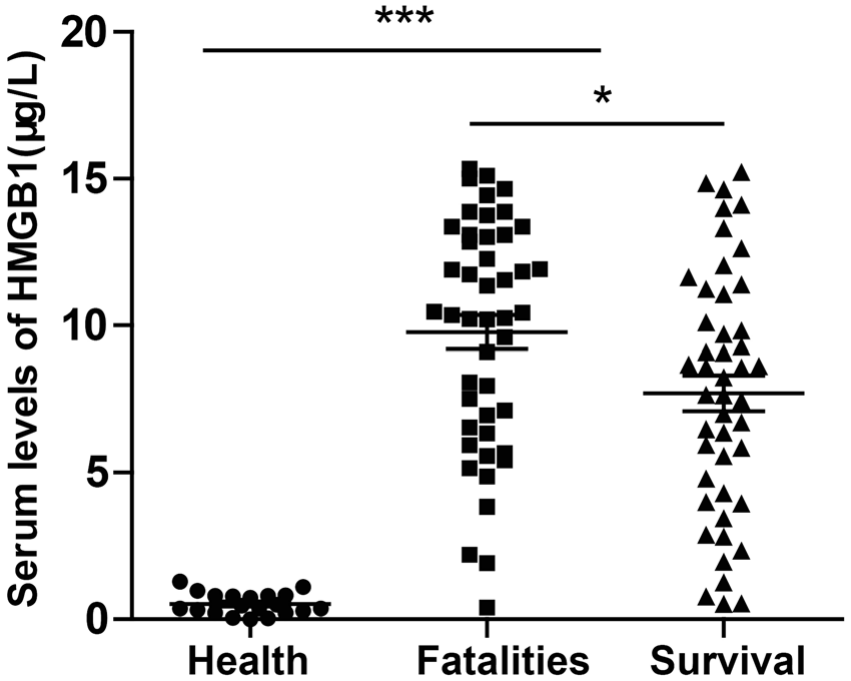
Comparison of serum HMGB1 levels in healthy controls, survivors and fatalities; **F** = **45.05**, *P* < 0.001; survivors and fatalities, t = **2.47**, *P* = 0.015; data are presented as mean ± SEM. ***P < 0.001, *P < 0.05.

Mean serum HMGB1 levels in healthy controls, fatalities, and survivors are shown in Fig. 1. Mean serum HMGB1 level in healthy controls was 0.53 [range, 0.00–1.30] μg/L, and significantly lower than for fatalities or survivors (*P* < 0.001). Mean baseline serum HMGB1 level was significantly higher in fatalities vs. survivors (11.79 [range, 0.41–15.34] μg/L) vs. 7.51 [range, 0.49–15.20] μg/L, *P* = 0.015) (Fig. 1).

### Comparison of the serum HMGB1 level in females and males

**Figure 2:**
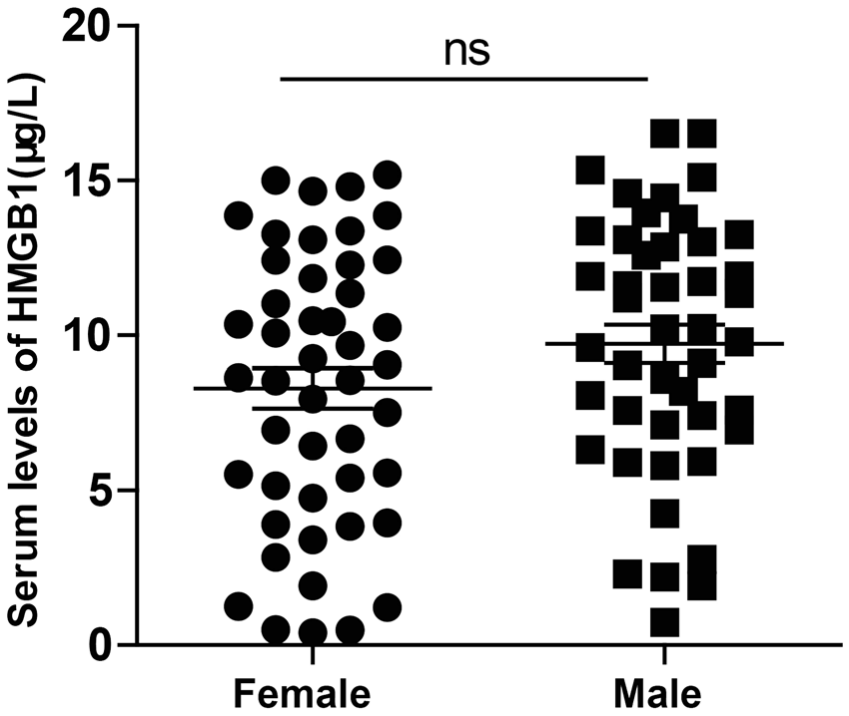
Comparison of serum HMGB1 levels in females and males; t = 1.60, *P* = 0.11. Data are presented as mean ± SEM; ns = not significant.

Mean serum HMGB1 levels in females and males are shown in Fig. 2. There was no significant difference in mean serum HMGB1 level between females and males (t = 1.60, *P* = 0.11) (Fig. 2).

### Association of serum HGB1 level with mortality rate

**Figure 3:**
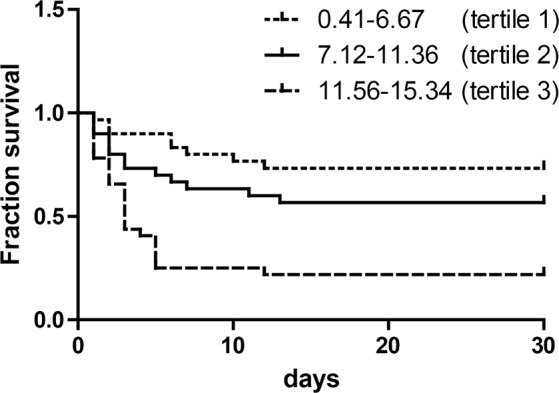
Kaplan-Meyer survival curve stratified by serum HMGB1 tertile (μg/L); χ^2^ = 21.86, *P* < 0.001 by the log-rank test.

All patients were treated according to standard procedures in the ER. Cumulative incidence of mortality during the first 30 days after admission was 50% (n = 46/92). The ratio of survivors to fatalities in each HMGB1 tertile is shown in Table 2. The 30-day mortality rate was significantly increased across tertiles from lowest (tertile 1, 26.7%; tertile 2, 43.3%) to highest (tertile 3, 78.1%) serum HMGB1 levels. A Kaplan-Meier curve was constructed to describe survival. The log rank test of a difference in survival between tertiles was significant (*P* < 0.001) (Fig. 3).

**Table 2:** 30-day mortality stratified by serum HMGB1 tertile.

HMGB1 Tertile	Survivors	Fatalities	30-days mortality (%)	χ^2^	*P*
Tertile 1	22	8	26.7		
Tertile 2	17	13	43.3	5.4	0.02*
Tertile 3	7	25	78.1	17.2	<0.001**

*tertile 1 vs. tertile 2.

**tertile 1 vs. tertile 2 vs. tertile 3.

### Risk factor analysis for 30-day mortality

Multivariate Cox proportional hazards regression analyses identified baseline serum HMGB1 level, WBC count, and serum lactic acid levels as independent prognostic markers of 30-day mortality in patients with acute paraquat poisoning (Table 3).

**Table 3:** Risk factors for 30-day mortality.

	Hazard ratio	95% confidence interval	*P*
HMGB1	1.635	1.086–2.460	0.018
lactic acid	1.726	1.086–2.743	0.021
WBC	1.508	1.024–2.223	0.038

HMGB1, high-mobility group box 1; WBC, white blood cell.

## Discussion

The results of the current study investigating acute paraquat poisoning in humans showed that 30-day mortality was increased in patients with higher baseline serum HMGB1 levels, and baseline serum HMGB1 level was an independent prognostic marker of 30-day mortality in this patient population.

Paraquat is a poisonous herbicide that has toxic effects due to its redox activity. Paraquat induces oxidative damage and cell death by generating ROS, including hydrogen peroxide, superoxide anion, and hydroxyl radicals.

HMGB1 in involved in infection, organ dysfunction and immune responses in acute injury and chronic disease, including myocardial infarction, stroke, acute lung injury, acute liver injury^7,9^, ischemia–reperfusion injury, sepsis, fibrotic disorders, and cancer^10^. HMGB1 is an alarmin, with distinct intracellular and extracellular effects. It is present in the cell nucleus and cytoplasm and can be translocated to the extracellular space^11^. HMGB1can be actively released from cells, including macrophages, monocytes, natural killer cells, dendritic cells, endothelial cells and platelets, or passively produced by necrotic and damaged cells^12,13^. In the extracellular space, HMGB1 mediates inflammation and chemotaxis^12^ and has far reaching effects causing remote organ dysfunction in sterile injury^14^.

Death in paraquat poisoning is usually caused by ALI^15,16^. The development and severity of ALI is controlled by pulmonary neutrophil infiltration^17–19^. Findings from our previous study revealed that the HMGB1-TLR4-I-L23-IL-17A axis regulated neutrophil infiltration and ALI in a mouse model of paraquat poisoning^7^. Conversely, administration of HMGB1-specific neutralizing antibodies or the HMGB1-binding antagonist thrombomodulin can rescue mice and rats^20–22^ from lethal sepsis. Taken together, data from our studies and previous animal experiments suggest that neutralization of HMGB1 represents a potential therapeutic option for the treatment of paraquat poisoning. However, the results should be interpreted with caution, as the specific mechanism of action of HMGB1 in humans remains to be elucidated.

The previous reports and findings from the current study suggest an association between HMGB1 and the mechanism of paraquat toxicity, in which HMGB1 may function as a regulator of cytokine, chemokine, and growth factor activity, coordinating the inflammatory and immune response^7,23^. Further investigations regarding this association are warranted to expand understanding of paraquat toxicity and inform the development of targeted therapeutics.

Some studies have determined that initial leucocyte level and arterial lactate are predictors of mortality in patients with paraquat poisoning^17,24–26^. This is consistent with our findings. Reliable predictors of prognosis are important to inform clinical treatment decision making in this patient population.

This study was associated with several limitations. First, this was a cross-sectional study and cause and effect of HMGB1 in the etiology of paraquat poisoning could not be elucidated. Second, oral paraquat poisoning was based on patient self- report. Evidence suggests that plasma paraquat levels have prognostic value in patients with acute paraquat poisoning^27^. However, plasma paraquat levels were not measured in the current study as access to testing is limited by time and cost, and most ER physicians do not rely on plasma paraquat measurements for clinical decision making.

In summary, the results of the current study suggest that serum HMGB1 level measured on admission to the ER is a feasible index for assessing the outcome of patients with acute paraquat poisoning, and strategies that decrease serum HMGB1 levels may have role in the management of these patients. Further studies elucidating the role of HMGB1 in paraquat poisoning are required to guide the development of effective clinical interventions.

## Supplementary information

serum HMGB-1 values for individual patients between fatalities and survivors

## Data Availability

The datasets generated during and/or analyzed during the current study are available from the corresponding author on reasonable request.
