# Supplementary material for: The significance of serum HMGB1 level in humans with acute paraquat poisoning
Source: Sci Rep. 2019 May 15;9:7448. doi: 10.1038/s41598-019-43877-1 (PMC6520495; doi:10.1038/s41598-019-43877-1)
Supplement: Supplementary file 1 — serum HMGB-1 values for individual patients between fatalities and survivors [file 41598_2019_43877_MOESM1_ESM.docx]

**Supplementary information**

**The significance of serum HMGB1 level in humans with acute paraquat poisoning**

Feng Chen ^1^, Zuolong Liu^2^, Wei Li^2^, Dan Li^3^*, Bailing Yan^2^*.

^1^Dermatology Department, China-Japan Union Hospital of Jilin University,126Xiantai Street, Changchun, Jilin Province, People’s Republic of China, 130033

^2^Department of Emergency, The First Hospital of Jilin University, 71Xinmin Street, Changchun, Jilin Province, People’s Republic of China, 130021

^3^Department of Respiratory Medicine, The First Hospital of Jilin University, 71Xinmin Street, Changchun, Jilin Province, People’s Republic of China, 130021

* These authors contributed equally to this work.

*Corresponding author:

Dan Li: Department of Respiratory Medicine, The First Hospital of Jilin University, 71Xinmin Street, Changchun, Jilin Province, People’s Republic of China, 130021, Email: [81945935@qq.com](mailto:81945935@qq.com).

Bailing Yan: Department of Emergency, The First Hospital of Jilin University, 71Xinmin Street, Changchun, Jilin Province, People’s Republic of China, 130021

Email:yanbailing@163.com.

**Supplementary data**

Table 1. Serum HMGB-1 values for individual patients between fatalities and survivors

| Serum HMGB-1 (μg/L) | | | |
| --- | --- | --- | --- |
| Fatalities | | Survivors | |
| 0.41 | 10.44 | 0.49 | 8.21 |
| 1.92 | 10.48 | 0.51 | 8.54 |
| 2.20 | 11.36 | 0.74 | 8.55 |
| 3.84 | 11.56 | 1.22 | 8.59 |
| 4.88 | 11.74 | 1.93 | 8.64 |
| 5.15 | 11.84 | 2.31 | 9.04 |
| 5.43 | 11.91 | 2.77 | 9.06 |
| 5.57 | 11.93 | 2.84 | 9.25 |
| 5.68 | 12.27 | 3.40 | 9.67 |
| 5.94 | 12.86 | 3.91 | 9.79 |
| 6.35 | 13.02 | 3.96 | 10.07 |
| 6.54 | 13.09 | 4.25 | 11.02 |
| 6.96 | 13.09 | 4.75 | 11.21 |
| 7.12 | 13.37 | 5.52 | 11.35 |
| 7.52 | 13.37 | 5.80 | 11.61 |
| 7.96 | 13.76 | 5.91 | 12.00 |
| 8.08 | 13.87 | 6.32 | 12.59 |
| 9.10 | 13.87 | 6.44 | 13.28 |
| 9.60 | 14.44 | 6.67 | 13.96 |
| 10.21 | 14.66 | 6.96 | 14.08 |
| 10.23 | 15.01 | 7.42 | 14.59 |
| 10.26 | 15.11 | 7.60 | 14.80 |
| 10.35 | 15.34 | 7.61 | 15.20 |
|  |  |  |  |
